# Dynamics of CD4 and CD8 T-Cell Subsets and Inflammatory Biomarkers during Early and Chronic HIV Infection in Mozambican Adults

**DOI:** 10.3389/fimmu.2017.01925

**Published:** 2018-01-05

**Authors:** Lucía Pastor, Victor Urrea, Jorge Carrillo, Erica Parker, Laura Fuente-Soro, Chenjerai Jairoce, Inacio Mandomando, Denise Naniche, Julià Blanco

**Affiliations:** ^1^AIDS Research Institute-IrsiCaixa, Hospital Germans Trias i Pujol, Badalona, Spain; ^2^ISGlobal, Barcelona Centre for International Health Research (CRESIB), Hospital Clínic–Universitat de Barcelona, Barcelona, Spain; ^3^Institut Germans Trias i Pujol (IGTP), Hospital Germans Trias i Pujol, Universitat Autonoma de Barcelona, Badalona, Spain; ^4^Centro de Investigação em Saúde da Manhiça (CISM), Maputo, Mozambique; ^5^School of Paediatrics and Child Health, University of Western Australia, Perth, WA, Australia; ^6^Universitat de Vic—Universitat Central de Catalunya, Vic, Spain

**Keywords:** AIDS, HIV pathogenesis, T-cell exhaustion, T-cell activation, immunosenescence, cytokines, acute HIV infection, sub-Saharan Africa

## Abstract

During primary HIV infection (PHI), there is a striking cascade response of inflammatory cytokines and many cells of the immune system show altered frequencies and signs of extensive activation. These changes have been shown to have a relevant role in predicting disease progression; however, the challenges of identifying PHI have resulted in a lack of critical information about the dynamics of early pathogenic events. We studied soluble inflammatory biomarkers and changes in T-cell subsets in individuals at PHI (*n* = 40), chronic HIV infection (CHI, *n* = 56), and HIV-uninfected (*n* = 58) recruited at the Manhiça District Hospital in Mozambique. Plasma levels of 49 biomarkers were determined by Luminex and ELISA. T-cell immunophenotyping was performed by multicolor flow cytometry. Plasma HIV viremia, CD4, and CD8 T cell counts underwent rapid stabilization after PHI. However, several immunological parameters, including Th1-Th17 CD4 T cells and activation or exhaustion of CD8 T cells continued decreasing until more than 9 months postinfection. Importantly, no sign of immunosenescence was observed over the first year of HIV infection. Levels of IP-10, MCP-1, BAFF, sCD14, tumor necrosis factor receptor-2, and TRAIL were significantly overexpressed at the first month of infection and underwent a prompt decrease in the subsequent months while, MIG and CD27 levels began to increase 1 month after infection and remained overexpressed for almost 1 year postinfection. Early levels of soluble biomarkers were significantly associated with subsequently exhausted CD4 T-cells or with CD8 T-cell activation. Despite rapid immune control of virus replication, the stabilization of the T-cell subsets occurs months after viremia and CD4 count plateau, suggesting persistent immune dysfunction and highlighting the potential benefit of early treatment initiation that could limit immunological damage.

## Introduction

During primary HIV infection (PHI), many cells of the immune system show signs of extensive activation and a progressive loss of resting subsets (1). Several T-cell subsets can be defined by their specificity, surface phenotype, or degree of maturation, and any or all of these parameters can be affected by HIV infection (2). Prior to changes in T-cell subsets, as HIV viremia increases during PHI, there is a striking cascade response of pro-inflammatory cytokines, which has been referred to as the “cytokine storm” (3). Although many of the cytokines present are common inflammatory effectors, their study can shed light on key pathogenic pathways associated with disease progression (4–9).

Generally, untreated HIV infection is characterized by progressive CD4 T-cell depletion and CD8 T-cell expansion. The profound CD4 T-cell depletion is linked directly to the risk for opportunistic infections and mortality (10). Likewise, CD8 T-cell activation (11–14) and exhaustion (15, 16) have been observed to be strong correlates of disease progression. The subsequent alterations in immune homeostatic mechanisms may lead to a progressive loss of the naïve and memory T-cell pool, resulting in an imbalance in T-cell phenotypes (10, 11). Similarly, after HIV infection, accelerated aging of T cells or immunosenescence may occur due to the continuous highly productive viral replication and cell stimulation (11, 17). Importantly, immunosenescence has also been associated with risk of adverse clinical events in HIV-infected individuals (18).

Besides interfering in T-cell maturation, HIV infection also affects T-cell functional diversity. A switch from Th1 to Th2 has been widely described (19), as well as changes in Th17 and Treg cells. Th17 cells are CD4-T cells involved in epithelial barrier integrity and protection against extracellular pathogens (20). Th17 cells have been seen to be irreversibly depleted in the gut-associated lymphoid tissue (GALT) during the first stages of PHI and have only been preserved by prompt ART initiation (21). Regulatory CD4 T cells (Tregs) induce tolerance against self-antigens and prevent autoimmunity (22–24). Interestingly, some studies have reported an increased Treg frequency in lymphoid tissues during progressive HIV disease (25, 26) as shown for SIV infection (27, 28), while others have shown a gradual decline in Tregs in peripheral blood associated with increased immune activation (29–32).

The challenges of identifying PHI have resulted in a lack of critical information that constrains the development of therapeutic interventions (33). In this study, we provide a longitudinal characterization of different T-cell subsets and the expression of soluble inflammatory biomarkers over the first year after PHI in a cohort of Mozambican adults and compared these changes with chronically HIV-infected (CHI) and HIV-uninfected subjects. Additionally, we explore the association between the various T-cell phenotypes and the plasma biomarker levels at different stages of infection.

## Materials and Methods

### Study Population

The study population was enrolled between 2013 and 2014 at the Manhiça District Hospital (MDH) in the district of Manhiça, Southern Mozambique. The present analysis is a sub-study of a prospective cohort of primary HIV-infected adults enrolled and followed up for 12 months in the gastrointestinal biomarkers in acute-HIV infected Mozambican adults study (GAMA) (34).

### Ethics Statement

This study was approved by local institutional review boards at Barcelona Clinic Hospital (2011/6264) and by the Ministry of Health of Mozambique (461/CNBS/12). All methods were carried out in accordance with the relevant guidelines and regulations. Written informed consent was obtained from patients prior to participation.

### HIV Diagnosis and Clinical Follow-up

All study participants were over 18 years of age and residents of the established District Surveillance System study area. During the screening, subjects presenting to the outpatient clinic of MDH for non-specific febrile symptoms or voluntary HIV counseling and testing (VCT) were included in the PHI group if they were negative or indeterminate for rapid test serology and HIV-RNA positive for pooled-viral load (VL) testing (*n* = 85). A control population was established by random selection among HIV-uninfected and individuals were invited to attend a study visit 1 month after the screening date (*n* = 58). PHI individuals were followed up at seven consecutive visits 1, 2, 3, 4, 6, 9, and 12 months after the screening visit. Technical information and procedures regarding HIV diagnosis and monitoring, as well as the screening profile have been previously described (34). Additionally, adults with documented HIV diagnosis ≥12 months earlier attending routine scheduled outpatient visits for clinical management of HIV/AIDS at the MDH were enrolled as CHI patients. CHI patients were included in the CHI-naïve or the CHI-ART, depending whether they had previously initiated treatment according to the current national guidelines (patients with a CD4 T-cell count ≤ 350 cells/mm^3^ or presenting and AIDS-associated disease).

After screening, demographic and clinical data was collected, medical consultation and HIV counseling was provided, and blood and stool samples were collected at all the study visits. Determination of CD4 and CD8 T-cell counts was performed on fresh whole blood in a single platform system using Trucount tubes and a FACScalibur flow cytometer. Peripheral blood mononuclear cells (PBMCs) were isolated by Ficoll density gradients and immediately stored in liquid nitrogen. VL determination was performed in plasma samples as previously described (34). Microbiological evaluation was performed in plasma and stool samples, testing for the most prevalent infections in the area including malaria, hepatitis B virus, syphilis and gastrointestinal protozoa, bacteria, and parasites (File S1 in Supplementary Material).

### Definition of PHI Phases and Quantification of Biomarkers

HIV-specific serology was subsequently performed on frozen plasma samples by western blot. VL and WB results at screening visit were employed to categorize individuals into Fiebig stages I–III (VL positive, WB negative), IV (VL positive, WB indeterminate), V (VL positive, WB positive with p31 band negative), and VI (VL positive, WB positive with p31 band positive) as described in previous work (34, 35). In order to approximate similar time since infection for the PHI individuals at every study visit, visits from individuals categorized in Fiebig stage V and VI at screening were moved 1 and 2 months forward, respectively, according to estimated days post infection previously described (35–37). After adjustment by Fiebig, new visits were grouped into M1, M2, M3, M4, M5, M6, M7–8, M9–11, and M12–15 according to estimated months since infection (Figure S1 in Supplementary Material). PBMCs or clinical data were not collected at the screening visit, so all the individuals included in M1 will not have this information, as well those individuals categorized in Fiebig stage V and VI at M2 and M3, respectively.

Multiplex biomarker profiling was performed for a total of 61 immune response biomarkers in plasma samples. Determinations were performed by ELISA commercial assays or Luminex technology as previously described (34–38). From the resulting 49 quantifiable biomarkers, the difference in the median levels between PHI and non-HIV-infected individuals with reported fever was highly significant for 13 soluble biomarkers (*P* < 0.001) (38). In order to represent the dynamics along the first year of infection, these 13 soluble biomarkers were grouped into two main pathways depending on their main function: (1) lymphocyte and monocyte function and (2) inflammation, intestinal damage, cell death, and proliferation.

### CD4 and CD8 T-Cell Immunophenotyping

Cryopreserved PBMCs were thawed at 37°C, washed in RPMI/60% and RPMI/20% of fetal bovine serum (FBS), and incubated for 1 h at 37°C in RPMI/10% FBS. PBMCs were then stained with the Fixable Viability Stain-FVS780r (APC-H7 detect, BD Biosciences) for 15 min. After PBMCs washing in PBS/1% FBS, cells were plotted to a U-bottom 96-well plate at a density of 1.5 millions/well and stained with selected 14-color panel including CD3-BV605 (Clone SK7), CD4-FITC (Clone RPA-T4), CD8-V500 (Clone SK1), CD45RA-Alexa Fluor^®^700 (Clone HI100), CD197-PE-CF594 (Clone 150503), CD57-APC (Clone NK-1), CD279-BV421 (Clone EH12.1), HLA-DR-BV650 (Clone G46-6), CD38-PerCp-Cy5.5 (Clone HIT2), CD25-PE (Clone M-A251), CD127-BV786 (Clone HIL-7R-M21), CD196-BV711 (Clone 11A9), and CD183-PE-Cy7 (Clone 1C6/CXCR3) (from BD Biosciences) for 15 min. After washing twice in PBS/1% FBS, cells were fixed in PBS/1% formaldehyde, acquired in a BD LSRFortessa cytometer using a plate HTS loader (BD Biosciences) and analyzed with FlowJo software (Tree Star). Gating strategy is described in Figure S2 in Supplementary Material. Lymphocyte gate was defined manually by morphological parameters excluding nonviable cells and singlets. Median of viability + lymphocytes was 2.5% [IQR 1.6–4.1], as an estimation of death cells per sample. Subsets were identified as CD3+ cells and gated as CD4+CD8− or CD8+CD4−, while double-positive cells and double-negative cells were excluded from the analysis. T-cell maturation stage was analyzed automatically using R software for CD45RA and CD197/CCR7 expression to define naive (TN, CD45RA+CCR7+), central memory (TCM, CD45RA−CCR7+), effector memory (TEM, CD45RA−CCR7−) and effector memory RA+ cells (TEMRA, CD45RA+CCR7−). T-cell subsets were also analyzed automatically for the expression of HLA-DR and CD38 to define activated cells (HLA-DR+ and CD38+), CD279/PD-1 to define exhausted cells (CD279+) and CD57 to define immunosenescent cells (CD57+). CD4+ T-cells were subsequently analyzed manually for the expression of CD25 and CD27 to define Treg subset (CD25+++CD27−) and automatically for the expression of CD183 and CD196 to define Th1/Th17 cells (CD183+CD196+).

### Statistical Analysis

Group comparisons were performed using the Fisher’s exact test for categorical variables and the non-parametric Kruskal–Wallis test for continue variables. Spearman’s correlation was used to assess the strength of relationship between continuous variables and multiple testing was further adjusted by false discovery rate. Individual comparisons between the different groups were performed using *post hoc* pairwise comparisons with the Tukey and Kramer (Nemenyi) test. Relative changes (*Z*-score) with respect to the HIV-uninfected group (in the case of VL the *Z*-score was calculated relative to the CHI-naïve group) have been represented by a transformation of the fitted longitudinal models by subtracting the mean and dividing by the standard deviation of HIV-non infected distribution, after a logarithmic transformation in the cases where it was required for normal distribution. Longitudinal behavior for analytes and immunological variables were modeled by fitting smoothing-splines mixed-effects models using the “sme” package of R. To infer if there was a significant association of selected biomarkers with the time variable, polynomial time effects approximation until third degree were fitted using linear mixed-effects regression models. Best model was selected based on likelihood ratio tests under maximum likelihood models estimations. A two-phase exponential decay regression model was employed in the case of VL modeling. Statistical analyses were performed using R-3.3.1 and Stata14 software.

## Results

### Characteristics of the Study Population

In the context of this study, we recruited 57 PHI patients identified during the screening process, as described in Section “Materials and Methods.” From these, 40 individuals attended a follow-up visit 1 month later and 26, 21, 14, 14, 13, and 11 of these patients continued visits at 2, 3, 4, 6, 9, and 12 months after screening, respectively. There was no significant difference in age, gender balance, or HIV-RNA VL between PHI patients who returned for enrollment and those who were lost to follow-up (34). HIV-uninfected individuals were randomly selected from screened individuals and 58 subjects representing the control population attended a visit 1 month later. During the cross-sectional recruitment of CHI individuals, 26 patients were included in the ART-naïve group and 30 patients in the ART group. The demographic and clinical characteristics of the 40 PHI individuals who started follow-up, the HIV-uninfected and the CHI groups, are summarized in Table 1. Significant differences were found for age and body mass index (BMI, *P* < 0.0001) but no differences were found for the clinical variables between the study groups (*P* > 0.05).

**Table 1:** Clinical and demographic characteristics of study population according to HIV-status.

	1st follow-up visit primary HIV infection (*n* = 40)	HIV-uninfected (*n* = 58)	CHI-naïve (*n* = 26)	CHI-ART (*n* = 30)	*P*-value
Age (years) [mean (SD)]	27.2 (9.2)	27.9 (9.5)	38.2 (13.4)	42.9 (8.8)	0.0001^a^
Gender [F (%)]	24 (60.0%)	46 (79.3%)	19 (73.1%)	19 (63.3%)	0.162^b^
Body mass index (kg/m^2^) [mean (SD)]	20.3 (3.1)	21.5 (4.1)	24.5 (4.6)	24.1 (3.2)	0.0001^a^
Time on ART (years) [median (IQR)]	–	–	–	2.6 (0.9–4.5)	–
Pregnant [*n* (% F)]	3 (12.5%)	7 (15.2%)	0 (0%)	3 (15.8%)	0.348^b^
Fever last 24 h [*n* (%)]	5 (12.5%)	3 (5.3%)	4 (15.4%)	1 (3.3%)	0.246^b^
Intestinal complaint last week [*n* (%)]	12 (30%)	15 (25.9%)	4 (15.4%)	2 (6.7%)	0.067^b^
Co-infections [*n* (%)]^c^					
Hepatitis B	5 (12.5%)	2 (3.5%)	2 (7.7%)	3 (10.0%)	0.400^b^
Syphilis	3 (7.5%)	4 (6.9%)	0 (0%)	1 (3.3%)	0.481^b^
Malaria	2 (5%)	0 (0%)	1 (3.9%)	0 (0%)	0.242^b^
Intestinal infection	6 (15%)	11 (19%)	2 (7.7%)	3 (10.0%)	0.552^b^

*^a^Comparisons of continuous variables were performed by Kruskal–Wallis test*.

*^b^Comparisons for proportions were performed by Fisher exact test*.

*^c^Co-infections were assessed as described in Supplementary Methods in Supplementary Material*.

**Figure 1:**
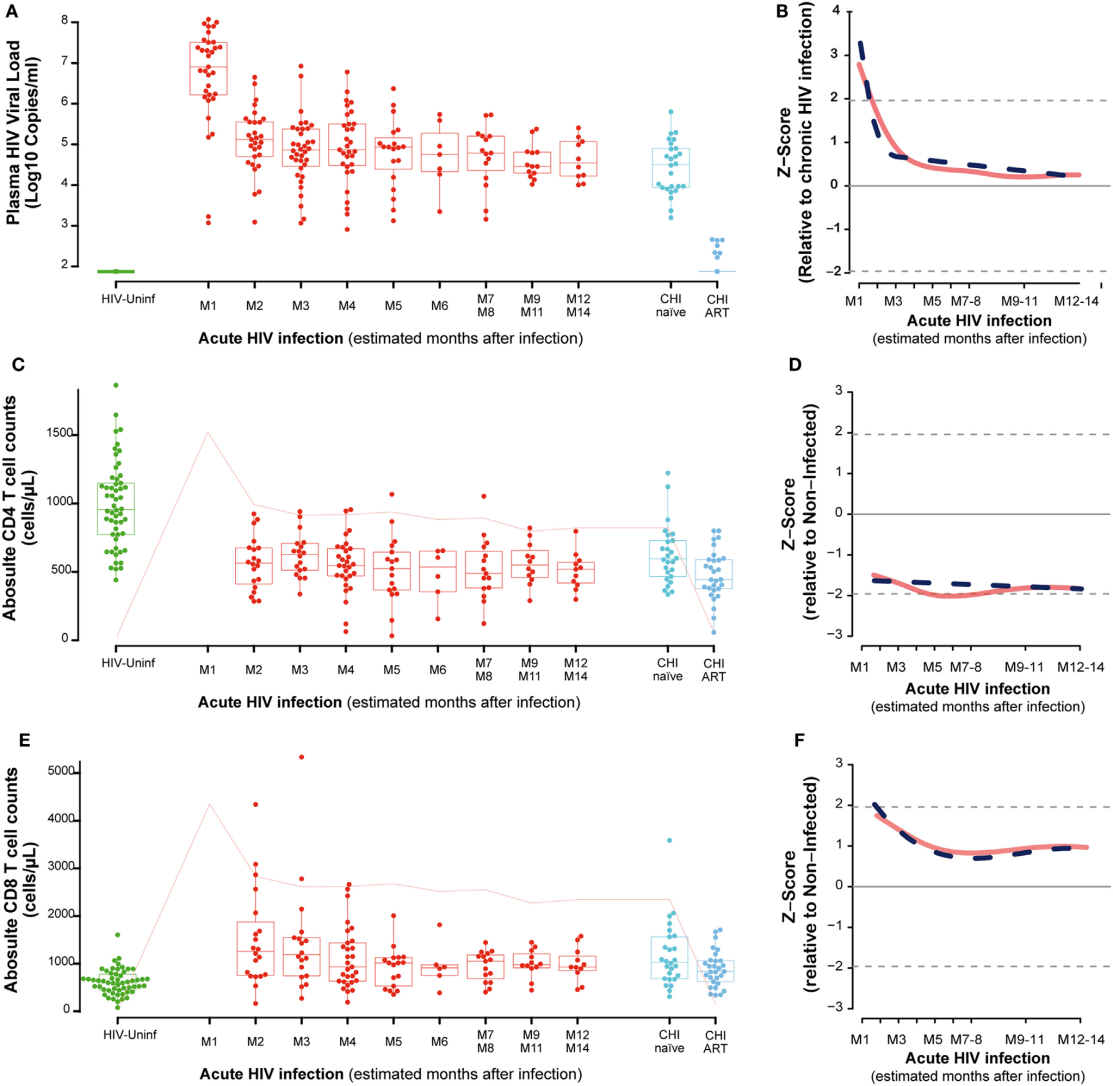
Virological and immunological characteristics along HIV infection. Plasma viral load (VL) as RNA Log10 copies/mL **(A)**, whole blood CD4 absolute count **(C)**, and whole blood CD8 absolute count **(E)** across the different study groups and along time postinfection. M, months after infection. Box as IQR, middle line as median, whiskers as maximum and minimum, and dots as individual observations in panels **(A,C,E)**. Pink line in panels **(C,E)** represents median VL at each time point for reference. Dynamics of each parameter **(B,D,F)** are shown as *Z*-score values for primary HIV infection individuals over CHI-naïve (VL) and over HIV-uninfected individuals (CD4 and CD8 T-cell counts). Red lines show non-parametric models, while dotted blue lines indicate the best fitting for polynomial time effects regression approximation.

Among the 57 PHI identified, 28, 5, 7, and 17 were categorized into Fiebig I-III, Fiebig IV, Fiebig V, and Fiebig VI stages, respectively, and were adjusted for time since infection as described in Section “Materials and Methods” (Figure S1 in Supplementary Material). After categorizing by Fiebig stage, and adjusting for time since infection, median VL in the PHI group was 6.9 RNA Log10 copies/mL (IQR 6.2–7.5) at month one (M1) and significantly decreased to 5.1 RNA Log10 copies/mL (IQR 4.7–5.6) at month 2 postinfection (M2), (*P* = 0.0001, Figure 1A). In the CHI-naïve patients, median VL was 4.5 RNA Log10 copies/mL (IQR 3.9–4.9).

In order to evaluate the dynamics of the different parameters along the first year of HIV infection, two approaches based on non-parametric modeling and linear regression modeling were performed as described in Section “Materials and Methods.” Longitudinal analysis confirmed the rapid decrease in VL with either a non-parametric models or a nadir VL set point. A biphasic exponential decay model revealed a second phase of VL decay with a lower but significant slope until 6 months after infection (M6, Figure 1B).

Regarding the dynamics of CD4 and CD8 T cells from M2, we observed that at M2 median CD4 T-cell count in PHI individuals was significantly lower than in the HIV-uninfected group, 565 (IQR 387–675) vs. 955 (IQR 773–1,149) cells/mm^3^, respectively (*P* = 0.0001, Figure 1C). CD4 T cells also showed an initial decrease that stabilized at months 5–6 postinfection (M5–6) in non-parametric longitudinal analysis, while significant linear decay was observed overtime in the regression model (*P* = 0.033 for the slope, Figure 1D). Median CD8 T-cell count was significantly higher in PHI at M2 than in HIV-uninfected controls, 1,175 (IQR 771–1,683) vs. 591 cells/mm^3^ (IQR 417–746), respectively (*P* = 0.0001, Figure 1E). Both longitudinal models show that the initial increase in CD8 T cells is followed by a significant decay that also stabilized at M5-6, remaining stable and high until CHI (*P* = 0.002, Figure 1F). In the CHI-naïve group, median CD4 T-cell and CD8 T-cell count were 595 (IQR 466–729) and 1,029 (IQR 685–1,562), respectively, while in the CHI-ART group, median CD4 T-cell and CD8 T-cell count were 474 (IQR 377–590) and 830 (IQR 617–1,061), respectively.

### CD4 Th1Th17 and Treg Changes during the Different Stages of HIV Infection

**Figure 2:**
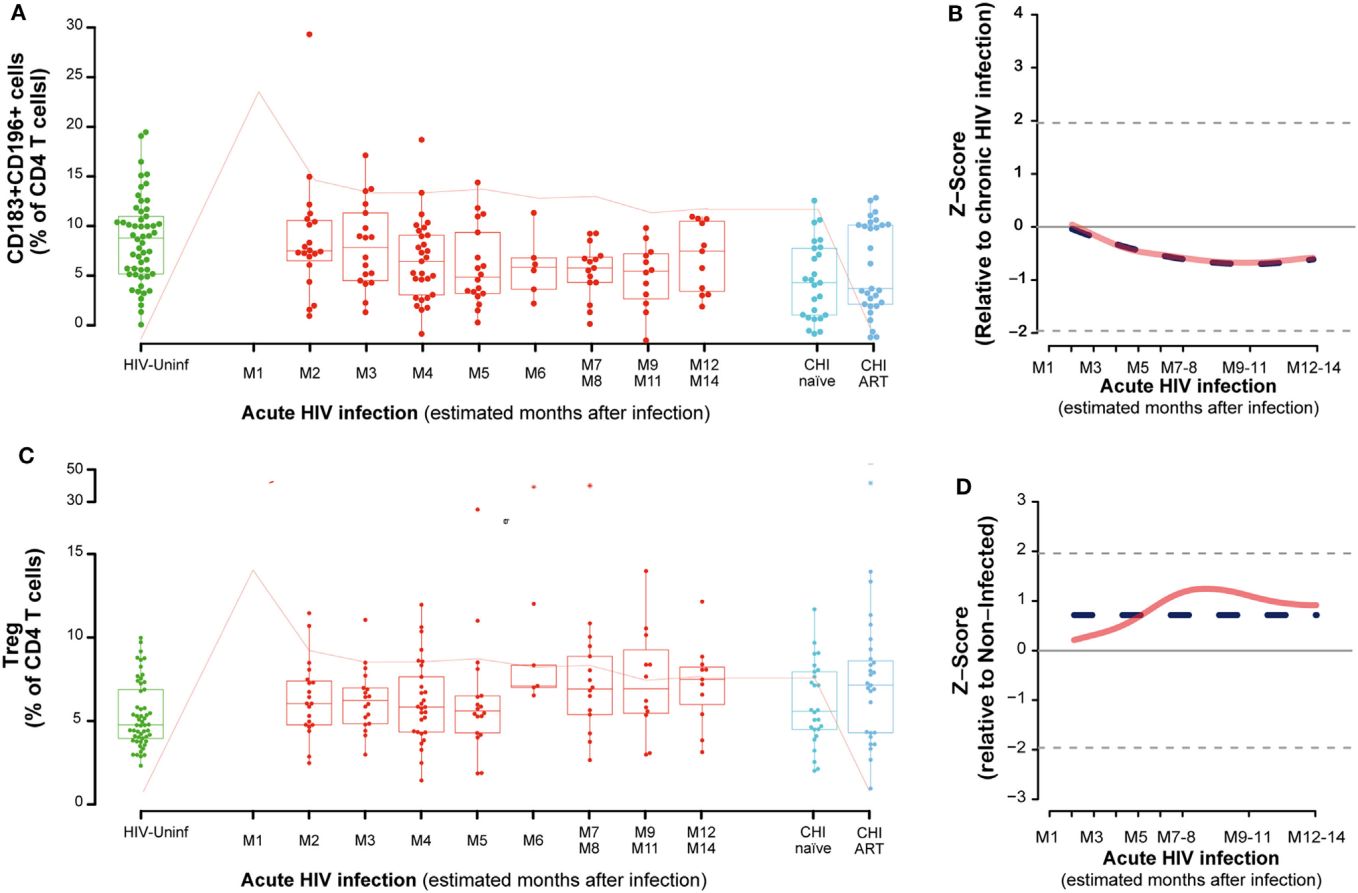
Dynamics of CD4 T-cell phenotypes along HIV infection. Characterization of Th1-Th17 **(A)**, Tregs **(C)** across the different study groups and along time postinfection. M, months after infection. Box as IQR, middle line as median, whiskers as maximum and minimum, and dots as individual observations. Pink line represents median VL at each time point for reference. Dynamics of each parameter **(B,D)** are shown as *Z*-score values for acutely infected individuals. Red lines show non-parametric models, while dotted blue lines indicate the best fitting for polynomial time effects regression approximation.

The frequency of functionally distinct CD4 T cells was analyzed by the cell surface expression of CD127 and CD25 (for Treg) and CD183 (CXCR3) and CD196 (CCR6) as described in Section “Materials and Methods.” This latter combination identifies Th1Th17 cells as CD183+CD196+, while CD183+CD196− cells are mostly Th1 and CD183− CD196+cells contain the Th17 population (Figure S2 in Supplementary Material) (19). No major changes were observed in CD4 CD183+CD196− or CD183−CD196+ cells during PHI (data not shown). However, the frequency of CD183+CD196+ (Th1Th17 cells) cells in PBMCs significantly decayed overtime until 7–8 months postinfection (M7–M8, *P* = 0.0127, Figures 2A,B). The percentage of activation in Th1Th17 cells at M2 (as measure by CD38 and HLA-DR co-expression) was significantly increased compared to HIV-uninfected (*P* < 0.0001) and continued to increase along the first year postinfection (data not shown). Looking at the maturation stage of the Th1Th17 cells, a significant increase in the frequency of naive (TN, *P* = 0.0002) and a significant decrease in the frequency of effector memory (TEM, *P* = 0.0007) was observed at M2 compared to HIV-uninfected. Since the definition of Th1Th17 cells involves cell surface expression of CXCR3, the receptor for IP-10, we assessed the relationship between CXCR3 expression and IP-10 levels. Although a significant negative correlation was observed between IP-10 plasma levels and circulating CD4 T cells at M2 (rho = −0.49, *P* = 0.0282); such association was positive and borderline significant between plasma IP-10 levels and CXCR3 + CD4 T cell frequencies (rho = 0.43, *P* = 0.0574), and no association was observed between plasma IP-10 and the frequency of Th1Th17 CD4 T cells. This fact could be explained because the kinetics of CXCR3+ and Th1Th17 CD4 T cells (CXCR3+CCR6+) are different, considering that expression of CCR6 has been reported to increase susceptibility to HIV infection (39).

No significant oscillations were observed during PHI for the CD4 Tregs (Figure 2C), but comparing to non-HIV infected individuals, CD4 Treg frequency was significantly higher in the CHI-ART group (*P* = 0.0267). Consistently, regression models showed no significant difference of Treg levels during the first year postinfection (Figure 2D).

### Intensive Loss of Resting CD8 Subsets Early after HIV Infection

**Figure 3:**
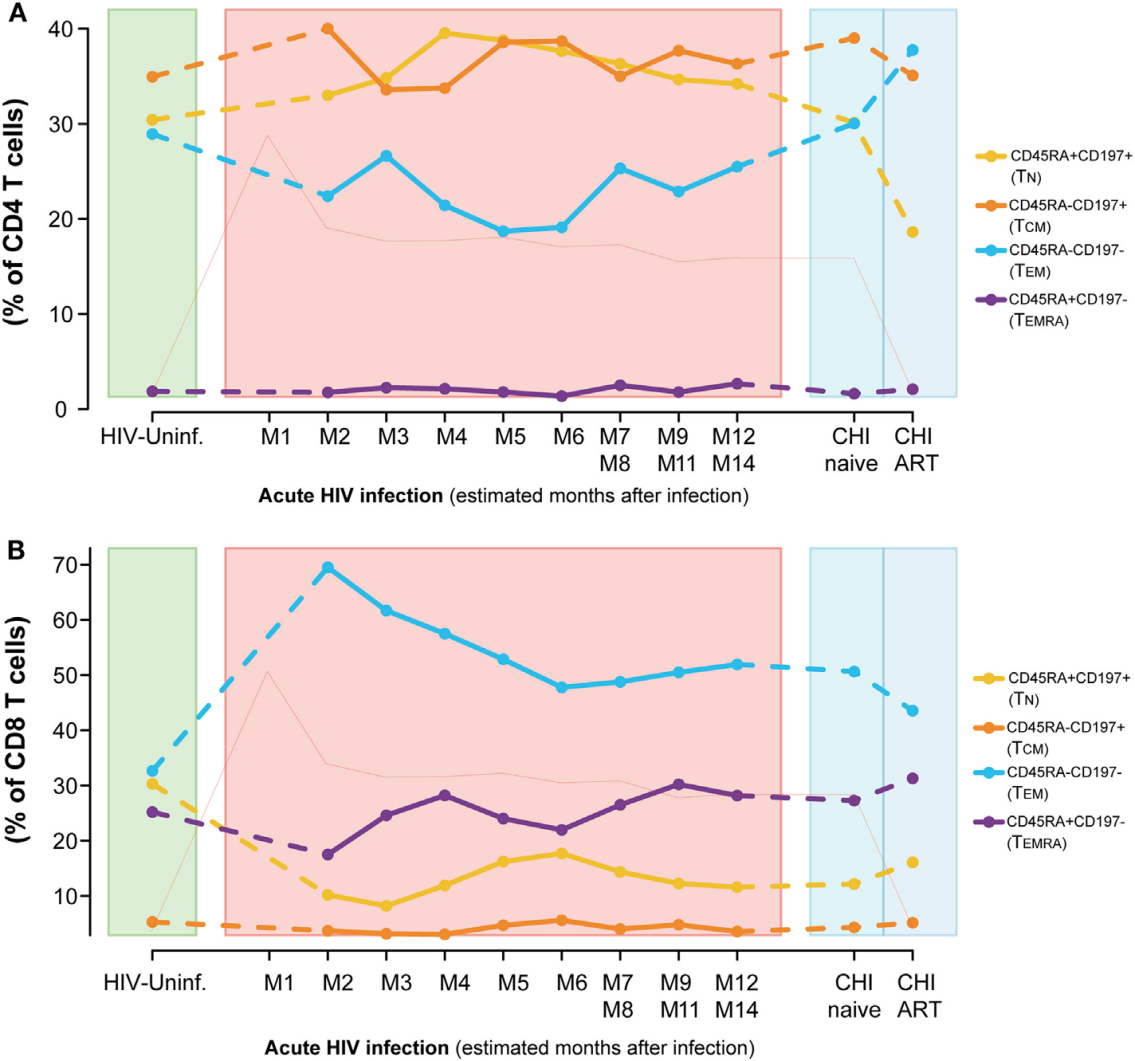
Dynamics of T-cell maturation phenotypes along HIV infection. Characterization of TN, TCM, TEM, and TEMRA frequencies for CD4 **(A)** and CD8 T cells **(B)** across the different study groups and along time postinfection. M, months after infection. Dot as median. Pink line represents median VL at each time point for reference.

Dynamics of the CD4 and CD8 T-cell maturation subsets showed a different profile. Although differences were not significant, the CD4 T-cell compartment showed a prompt increase in the resting phenotypes (TN and central memory T cells [TCM]) and a decrease in the effector phenotypes [TEM and effector RA+ T cells ([TEMRA)] at M2 (Figure 3A) compared to HIV-uninfected, that normalized several months after infection. These longitudinal changes observed in the CD4 T-cell compartment over the first year of infection, were not significant for any subset. When comparing CHI-naïve and CHI-ART groups, CHI-ART group showed a non-significant tendency toward higher levels of TEM and lower significant levels of TN (*P* = 0.0413).

Conversely, we observed a marked loss of the TN and TCM CD8 T-cell pool after HIV infection concomitant to an increase in the frequencies of the TEM subset (Figure 3B). Comparing to HIV-uninfected individuals, CD8 TN significantly decreased in PHI at M2 and in CHI-naïve groups (*P* < 0.0001) and CD8 TEM significantly increased (*P* < 0.0001, *P* = 0.0002, respectively). No significant changes were observed in TCM; however, TEMRA was significantly increased in CHI-ART compared to PHI at M2 (*P* = 0.0027). Longitudinal analysis confirmed these changes (data not shown).

### T-Cell Activation Phenotypes along HIV Infection

The dynamics of activation (CD38+ HLA-DR+ cells) exhaustion (CD279+ cells) and immunosenescence (CD57+ cells) were also analyzed in CD4 and CD8 T cells. As for maturation markers, most relevant changes were noticed in CD8 T cells. Although both activated and exhausted CD4 T cells were significantly increased at M2 when compared to HIV-uninfected individuals (*P* < 0.0001, *P* = 0.0056, respectively), and slowly but significantly decayed over the first year of infection (Figures S3A,B in Supplementary Material). Immunosenescent CD4 T cells (CD57+) showed no significant changes in the PHI group as compared to HIV-uninfected individuals but they were significantly increased in the CHI-ART group (*P* = 0.0001, Figure S3C in Supplementary Material).

**Figure 4:**
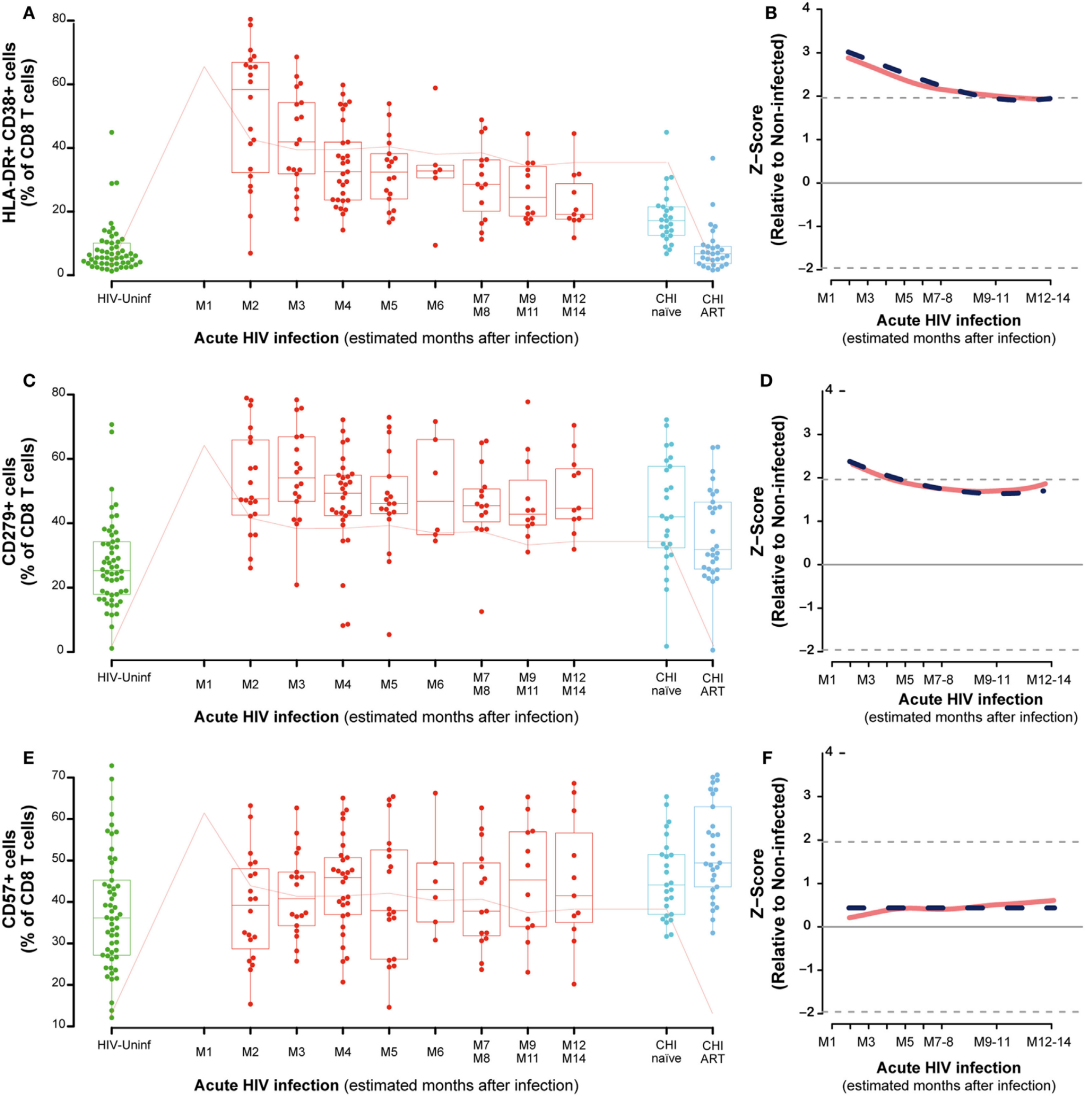
CD8 T-cell activation, exhaustion, and immunosenescence along HIV infection. Characterization of activated **(A)**, exhausted **(C)**, and senescent CD8 T-cells **(E)** across the different study groups and along time postinfection. M, months after infection. Box as IQR, middle line as median, whiskers as maximum and minimum and dots as individual observations. Pink line in panels **(A,C,E)** represents median VL at each time point for reference. Dynamics of each parameter **(B,D,F)** are shown as *Z*-score values for acutely infected individuals. Red lines show non-parametric models, while dotted blue lines indicate the best fitting for polynomial time effects regression approximation.

The analysis of activation in CD8-T cells showed a significant increase at M2 as compared to HIV-uninfected that remained in CHI-naïve subjects (*P* < 0.0001) and normalized in CHI-ART (Figure 4A). The CD8 T-cell activation observed in the first months of infection was significantly reduced over time with a slow but significant decrease until months 9–11 (M9–11) (*P* < 0.0001, Figure 4B). Exhausted CD8 T cells also showed a transient but less marked significant increase as compared to HIV-uninfected individuals (*P* < 0.0001, Figure 4C), with a significant and slow decrease over time (*P* = 0.0056, Figure 4D). Along the course of HIV infection, the frequency of senescent CD8 T-cells was significantly higher in the CHI-ART group as compared to the HIV-uninfected group (*P* < 0.0001, Figure 4E). However, no significant changes over time were observed in the frequency of senescent CD8 T-cells by linear regression models (Figure 4F). There were no significant differences in percentages of activated, exhausted or senescent CD8 T-cells associated with the presence of co-infection in any of the study groups.

### Dynamics of Soluble Inflammatory Biomarkers and Association with T-Cell Phenotypes in PHI

**Figure 5:**
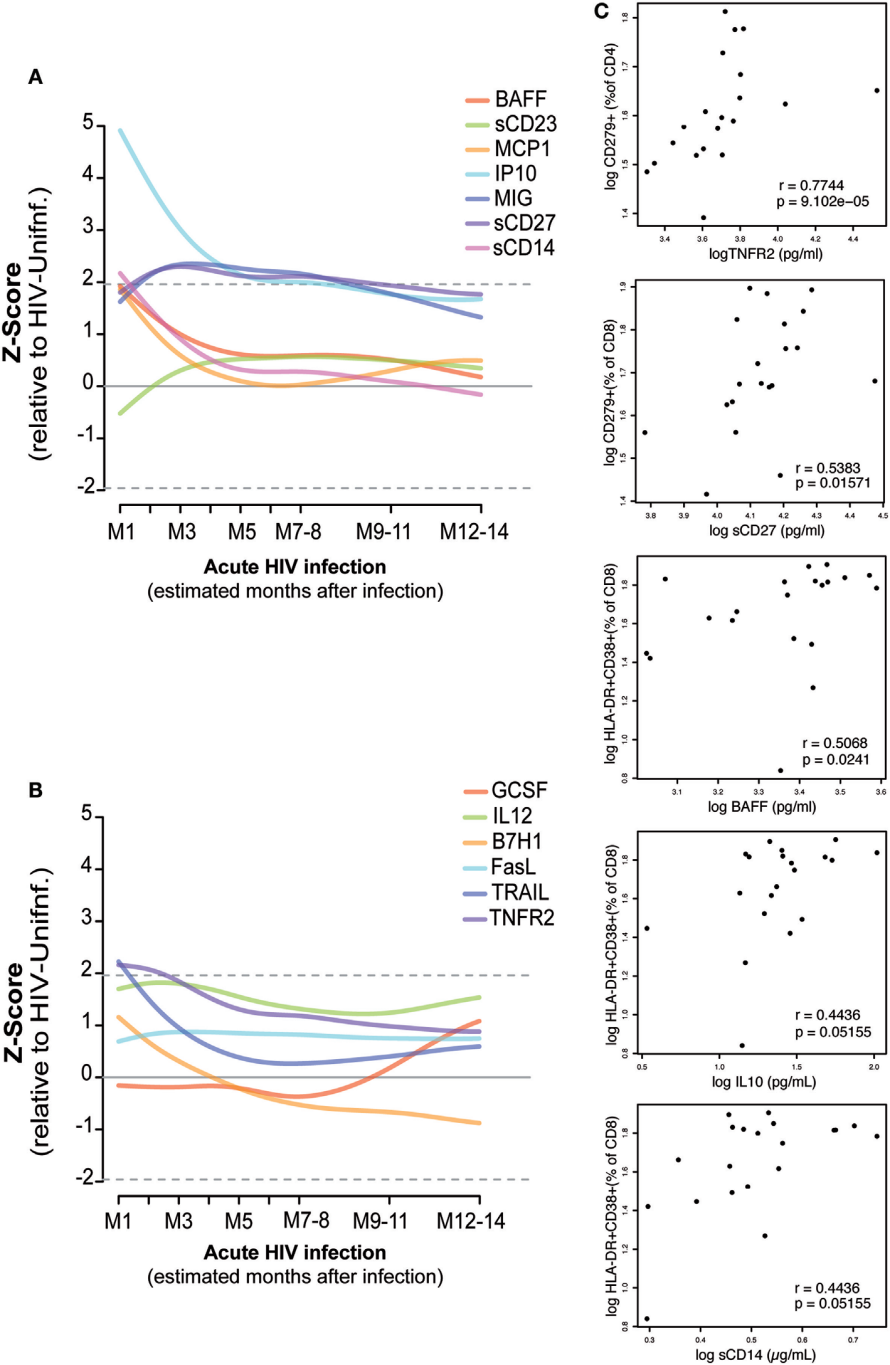
Association of plasma biomarker levels and T-cell phenotypes during early HIV infection. Biomarker normalized expression levels (*Z*-score relative to HIV-uninfected controls) along the first year postinfection **(A,B)**. Biomarkers showing significant correlation at one month postinfection with the subsequent exhausted or activated T-cell phenotypes at 2 months postinfection are shown in panel **(C)**. Spearman rho correlation and *P*-value are shown for each plot.

As described in Section “Materials and Methods,” the kinetics of 13 soluble biomarkers with expression levels most significantly different between febrile PHI and HIV-uninfected individuals (38) were characterized in detail during the first year after infection (Figures 5A,B; Figure S4 in Supplementary Material). Levels of IP-10, MCP-1, BAFF, soluble (s)CD14, tumor necrosis factor receptor-2 (TNFR2), and TRAIL were highly overexpressed at the first month of infection (M1) and underwent a prompt decrease in the subsequent months. This decrease was more gradual in the case of IP-10, which remained overexpressed even 5 months postinfection. On the contrary, MIG, sCD27, and sCD23 levels started to increase 1 month after infection and remained overexpressed for almost 1 year postinfection, while GSCF had a later upregulation at 7 months of infection.

Correlations between the plasma biomarker levels at M1 and activated or exhausted CD4 and CD8 T-cell phenotypes at 2 months postinfection were assessed as described in Section “Materials and Methods” (Figure 5C). We observed a significant positive correlation between M1 TNFR2 and sCD27 levels and the frequency of exhausted CD4 T cells and CD8 T cells at M2 (rho = 0.77, *P* < 0.0001; rho = 0.54, *P* = 0.0157, respectively). Similarly, we saw a significant association between M1 levels of BAFF (rho = 0.50, *P* = 0.0241), IL10 (rho = 0.44, *P* = 0.05), and sCD14 (rho = 0.44, *P* = 0.05) and the frequency of activated CD8 T-cells at M2. However, after adjustment by multiple testing, only the significance of TNFR2 with exhausted CD4 T-cells levels was maintained (*P* = 0.0135). We did not observe significant differences in the expression level of these 13 selected soluble biomarkers by the presence of any co-infection in the PHI or the CHI groups; however, BAFF, MCP-1, MIG, and TRAIL expression levels were significantly lower among the individuals included in the HIV-uninfected control group with a co-infection detected (*P* = 0.0254, *P* = 0.0418, *P* = 0.0001, *P* = 0.0032, respectively).

## Discussion

We conducted a systematic analysis of the clinical, virologic, and immunologic characteristics of the different stages of HIV infection in HIV-infected Mozambican adults. Soluble biomarker quantification and T-cell immunophenotyping revealed that while most inflammatory biomarkers, CD4 counts and VL stabilized early after HIV infection, certain T-cell subsets took longer to reach a stable level.

Several studies have shown that during AHI up to 80% of CD4 memory T-cells in GALT is destroyed within the first 3 weeks of infection (40–42). Particularly, depletion of memory CD4 Th17-cells in GALT occurs at the first stages of acute HIV infection (21). However, data from our PHI cohort show that these changes are not evident in circulating cells. For the specific case of systemic Th1Th17 cells, their frequency in PBMCs is similar to uninfected individuals at 2 months after infection but decreases steadily until 9–11 months after infection displaying and maintaining an activated phenotype early after infection. Since the definition of Th1Th17 cells involves cell surface expression of CXCR3, the receptor for IP-10, and this cytokine has been associated with the recruitment of CXCR3+CD4 T cells to HIV replication sites (43), we assessed the relationship between CXCR3 expression and IP-10 levels. Although a significant negative correlation was observed between IP-10 plasma levels and absolute numbers of circulating CD4 T cells, such association was not observed between plasma IP-10 and the frequency of CXCR3+ or Th1Th17 (CD183+CD196+) CD4 T cells. In contrast to Th17 cells, studies have reported both an increased (25, 26) and a gradual decline in Treg frequency in peripheral blood during progressive HIV infection (29–32). Although we did not observe significant changes in the Treg compartment during PHI, we detected a trend toward an increase after HIV infection.

Surprisingly, ART seemed to lack beneficial effects in restoring the CD4 T-cell maturation profile as the CHI-ART group showed higher levels of TEM and lower levels of TN than did CHI-naive. This is probably due to poor immunological recovery that associates with a skewed CD4 T cell maturation (44, 45).

CD8 T-cell activation has been described to be the strongest correlate of disease progression (11–14). Recently, the magnitude and kinetics of CD8 T-cell activation during early acute HIV infection has been observed to impact VL set point (46). Still, we observed in our study that both activated and effector memory CD8 T-cells peaked at month 2 after infection and reached stable levels only at 9–11 months postinfection, several months after viremia stabilization. These results indicate that, despite the rapid immune control over virus replication, homeostasis in the CD8 T-cell compartment requires longer to be achieved. Thus, most alterations observed in the CD8 T-cell compartment during HIV infection are not exclusively viremia driven. Our data also show an early increase of the exhaustion marker PD-1 (CD279) that slowly decays paralleling activation in CD8 T cells. Importantly, despite these profound alterations, no relevant increases of the expression of CD57 were observed in CD8 T cells during PHI, suggesting that this marker of replicative immunosenescence could be associated with longstanding HIV infection, suggested by its highest expression in CHI individuals. Moreover, increased CD57 expression in CD8 T-cells was previously reported to be associated with age (47) and ART initiation (48).

Significant efforts have been made to characterize early cytokine responses with the aim of identifying biomarkers of progression or key pathological pathways that could be targeted to minimize HIV-induced immune damage (4–6, 49). In this study, we provide additional data showing associations between early soluble biomarker levels and specific T-cell phenotypes 2 months after infection. TNFR2 and sCD27 levels were associated with exhausted CD4 T-cells and CD8 T-cells, respectively, while BAFF, interleukin-10 (IL-10), and sCD14 were associated with CD8 T-cell activation. TNFR2 is involved in cell survival that can result in cell proliferation, while sCD27 participates in generation and long-term maintenance of T-cell immunity. Thus, by function, these two soluble biomarkers could reflect early activation of CD4 and CD8 T-cells after HIV infection that subsequently lead to a higher proportion of exhausted T-cells. B-cell activating factor (BAFF), IL-10, and sCD14 are produced by monocytes and macrophages after infection or tissue inflammation in order to assure a proper immune response (50, 51) so their association with the subsequent CD8 T-cell activation could indicate a way of controlling the cellular response to HIV infection.

Adjustment by Fiebig stage at the screening allowed us to approximate time since infection according to previous categorization (35–37). However, this approximation may add potential uncertainty to the biomarker levels detected during the first 3 months after infection when the very intense immune responses are occurring (35–37). Additionally, age and BMI were significantly higher in the CHI groups, comparing to the PHI and control HIV-uninfected population. This is explained by the high HIV-incidence rate in young population in the Sub-Saharan setting and the national ART recommendations in place at the moment of the study. According to the HIV guidelines in Mozambique in 2013–2014, HIV-infected patients initiated ART if CD4 T-cell counts were ≤350 cells/mm^3^ or presenting an AIDS-associated disease, features more common at late stages of HIV infection. This fact might impact immune recovery (52) and along with age and BMI differences could have affected the biomarker comparison with CHI groups, especially at the analysis of T-cell maturation stages (53), immunosenescence (11), and soluble biomarker expression (54, 55).

Due to the study design, T-cell immunophenotyping data were not available for the first month of infection. This would have allowed a further characterization of the first responses in the T-cell compartment and provided additional data in the T-cell specific phenotypes. Similarly, the loss to follow-up along the longitudinal visits may have resulted in insufficient power to detect additional significant differences. The high loss to follow-up also hampered the possibility to study the associations between soluble and cellular markers with clinical disease progression in our cohort. Such a high attrition rate is common in these scarce-resource rural settings (56). Attendance of scheduled visits is complicated by high rates of migration, long distances to health centers and difficulties missing work, which threat continuity of care. Moreover, PHI individuals are usually asymptomatic after peak viremia (57), so patients do not feel the need to return to the hospital until they have further progressed to AIDS. Additionally, some PHI individuals met criteria for ART initiation during the study, and therefore follow-up interruption, due to pregnancy or AIDS-associated conditions.

The high burden of infectious diseases prevalent in the study area could have impacted the T-cell phenotypic characteristics and soluble biomarker dynamics and expression levels. However, we did not observe any significant difference according to the co-infection status in the stage of CD8 activation for any of the study groups. We did find that soluble biomarker expression levels in those individuals who were positive for intestinal, malaria, hepatitis B, or syphilis infection were significantly higher for BAFF, MCP-1, MIG, and TRAIL as compared to those negative for all the tested infections, but only in the HIV-uninfected group. Thus, further studies could evaluate the specific effect that additional co-infections could have in the dynamics of these cellular and plasma biomarkers in the HIV-infected individuals. The soluble biomarker levels for PHI patients prior to onset of symptoms were not available. Our results thus describe the soluble biomarker levels after the start of the “cytokine storm” from at approximately 10 (95% CI 7–21) days postinfection (35–37), when VL and cytokine levels are already close or pass to their peak.

This characterization of biomarker expression in plasma and T-cells during the different stages of HIV infection provides an in depth description of the immune responses following HIV acquisition in a population of Mozambican adults. Several studies have provided description of the cytokine (3–9) or T-cell phenotypes (33, 57, 58) during acute and PHI. However, our longitudinal study offers new insight into potential associations between innate and cellular responses. Early ART stops progression to AIDS (59, 60), diminishes the size of viral reservoir (61), prevents intestinal damage (21), and reduces further transmissions (62). In our study, we also show that stabilization of specific T-cell phenotypes occurs months after viremia or CD4 count stabilize in the course of infection, adding more evidence to the arguments for treatment initiation regardless of CD4 counts or viremia levels. Previous studies have seen that ART initiation at the earliest stages of acute HIV infection does not normalize the CD4/CD8 ratio even after 2 years of treatment (33), suggesting some degree of persistent immunological dysfunction. Our data show that homeostasis in the CD8 T-cell compartment and initiation of Th1Th17 decay in PBMCs occurs months after viremia and CD4 count reach the set point level. This indicates that many HIV-related changes observed in the CD8 T-cell and CD4 T-cell compartment may not be exclusively driven by viremia levels and additional immune responses could account for these T-cell alterations. This raises the potential need for additional therapies that could enhance immune recovery and reduce immune activation.

## Ethics Statement

The study population was enrolled between 2013 and 2014 at the Manhiça District Hospital (MDH) in the district of Manhiça, Southern Mozambique. The present analysis is a sub-study of a prospective cohort of primary HIV-infected adults enrolled and followed up for 12 months in the gastrointestinal biomarkers in acute-HIV infected Mozambican adults study (GAMA) (34). This study was approved by local institutional review boards at Barcelona Clinic Hospital (2011/6264) and by the Ministry of Health of Mozambique (461/CNBS/12). All methods were carried out in accordance with the relevant guidelines and regulations. Written informed consent was obtained from patients prior to participation.

## Author Contributions

DN and JB study design. LP, EP, and LF-S recruited subjects and collected clinical data. LP, EP, LF-S, and CJ performed laboratory analysis at the field. LP and JC performed plasma biomarker quantification and validation of the data. LP, VU, and JB performed PBMCs phenotyping and validation of the data. LP and VU performed statistical analyses. LP, VU, DN, and JB interpreted the data. LP, EP, LF-S, IM, DN, and JB study management and coordination. LP drafted the paper. VU, DN, and JB critical data review and revision of manuscript writing. All authors read and approved the final version of the manuscript.

## Conflict of Interest Statement

The authors declare that the research was conducted in the absence of any commercial or financial relationships that could be construed as a potential conflict of interest.
