# Supplementary material for: Dynamics of CD4 and CD8 T-Cell Subsets and Inflammatory Biomarkers during Early and Chronic HIV Infection in Mozambican Adults
Source: Front Immunol. 2018 Jan 5;8:1925. doi: 10.3389/fimmu.2017.01925 (PMC5760549; doi:10.3389/fimmu.2017.01925)
Supplement: Supplementary file 1 [file Table_1.PDF]

Dynamics of CD4 and CD8 T-cell subsets and inflammatory biomarkers during early and chronic HIV infection in Mozambican adults. **L Pastor *et al*.**

## Supplementary Table 1. Summary of the polynomial models parameters.

| | Intercept | time | | time^2 | | time^3 | | |
|---|---|---|---|---|---|---|---|---|
| | | coef. | pval | coef. | pval | coef. | pval | LR test |
| CD4 count | 562,38 | -5,36 | 0,0349 | | | | | 0,0328 |
| ln(CD8 count) | 7,42 | -0,29 | 0,0019 | 0,04 | 0,0095 | 0,00 | 0,0231 | 0.0019 |
| CD4+CD183+CD196+ | 10,37 | -0,73 | 0,0116 | 0,04 | 0,0373 | | | 0,0127 |
| CD4+ | 7,16 | | | | | | | ns |
| CD8+CD45RA+CD197+ | 11,21 | 1,73 | 0,0021 | -0,11 | 0,0025 | | | 0,0089 |
| CD8+CD45RA-CD197+ | 4,03 | | | | | | | ns |
| CD8+CD45RA-CD197- | 65,07 | -3,42 | 0,0005 | 0,19 | 0,0042 | | | 0,0003 |
| CD8+CD45RA+CD197- | 21,95 | 0,66 | 0,0005 | | | | | 0,0005 |
| ln(CD8+HLADR+CD38+) | 3,94 | -0,15 | < 0.0001 | 0,01 | 0,0042 | | | < 0.0001 |
| ln(CD4+HLADR+CD38+) | 2,08 | -0,11 | 0,0044 | 0,01 | 0,0506 | | | < 0.0001 |
| CD8+CD279+ | 54,26 | -2,25 | 0,0094 | 0,12 | 0,0402 | | | 0,0055 |
| CD8+CD57+ | 42,13 | | | | | | | ns |
| CD4+CD279+ | 37,97 | -0,51 | 0,0001 | | | | | 0,0001 |
| CD4+CD57+ | 10,48 | | | | | | | ns |
| CD4+CD45RA+CD197+ | 36,08 | | | | | | | ns |
| CD4+CD45RA-CD197+ | 34,75 | | | | | | | ns |
| CD4+CD45RA-CD197- | 26,57 | | | | | | | ns |
| CD4+CD45RA+CD197- | 2,75 | | | | | | | ns |

ns: non significant time effect
