# Supplementary material for: Dynamics of CD4 and CD8 T-Cell Subsets and Inflammatory Biomarkers during Early and Chronic HIV Infection in Mozambican Adults
Source: Front Immunol. 2018 Jan 5;8:1925. doi: 10.3389/fimmu.2017.01925 (PMC5760549; doi:10.3389/fimmu.2017.01925)
Supplement: Supplementary file 2 [file Data_Sheet_1.PDF]

Supplementary Methods

Methodology employed for the screening of the most prevalent co-infections.

- Plasmodium falciparum malaria: Whole blood was used to screen for Plasmodium falciparum malaria using a glass slide for optic microscopic determination.

- Hepatitis B Virus (HBV): Rapid testing for HBV surface antigen was performed in whole blood by using the Determine assay (Inverness Laboratories) according to manufacturer's instructions.

- Treponema pallidum: Screening for syphilis was performed in plasma samples using the Rapid plasma reagin (RPR) according to manufacturer's instructions (Human Diagnostics). All rapid plasma reagin–positive samples were confirmed by a using a Treponema pallidum Hemagglutination (TPHA) assay (Human Diagnostics).

- Gastro-intestinal infections were evaluated in stool samples as:

  • Giardia Lamblia, Cryptosporidium sp. and Entamoeba Histolytica were assessed by ELISA immune sorbent assay.

  • Salmonella, Shigella spp, Vibrio cholerae, Aeromona, Yersinia were cultured and identified by selective mediums.

  • Parasitological infections were examined by optic microscopy.

  • Cryptosporidium difficile infection was detected using a commercial immunoassay, which detects oocyte antigen and according to manufacturer's instructions (C. diff quik chek complete test, Alere, TechLab).
