# Supplementary material for: Dynamics of CD4 and CD8 T-Cell Subsets and Inflammatory Biomarkers during Early and Chronic HIV Infection in Mozambican Adults
Source: Front Immunol. 2018 Jan 5;8:1925. doi: 10.3389/fimmu.2017.01925 (PMC5760549; doi:10.3389/fimmu.2017.01925)

Supplementary Figure 1. Study time points after adjustment by Fiebig. According to estimated months since infection (references 36–38 of the manuscript), new visits were grouped into M1, M2, M3, M4, M5, M6, M7-8, M9-11, and M12-14. S0, screening visit; V, visit; M, month; “X”, excluded visits

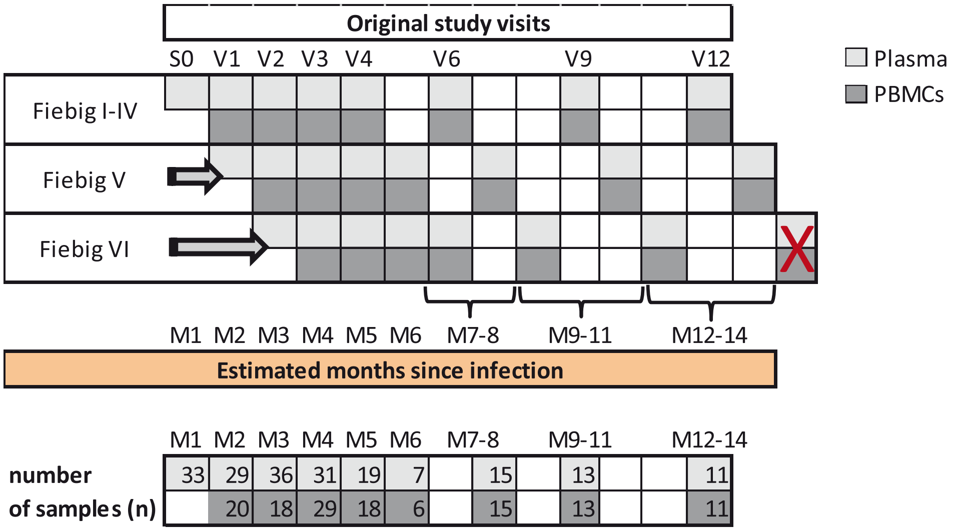

Supplement: Supplementary file 3 [file Image_1.PDF]
