# Supplementary material for: Dynamics of CD4 and CD8 T-Cell Subsets and Inflammatory Biomarkers during Early and Chronic HIV Infection in Mozambican Adults
Source: Front Immunol. 2018 Jan 5;8:1925. doi: 10.3389/fimmu.2017.01925 (PMC5760549; doi:10.3389/fimmu.2017.01925)

Dynamics of CD4 and CD8 T-cell subsets and inflammatory biomarkers during early and chronic HIV infection in Mozambican adults. **L Pastor *et al***

Supplementary Figure 2. Gating strategy for T-cell analysis. PBMCs were acquired and gated according to i) time (to ensure homogeneous acquisition), ii) FSC area and height (to select singlets), iii) SSC and FSC (morphology) and iv) to CD3 staining and viability to select for living T cells. Then CD4 and CD8 cells were identified. For CD8 and CD4 T cells, maturation, activation, exhaustion or senescence were defined as described in Methods. CD4+ T-cells were further analyzed for Treg frequency and for the expression of CXCR3 (CD183) and CCR6 (CD196) to define Th1/Th17 cells.

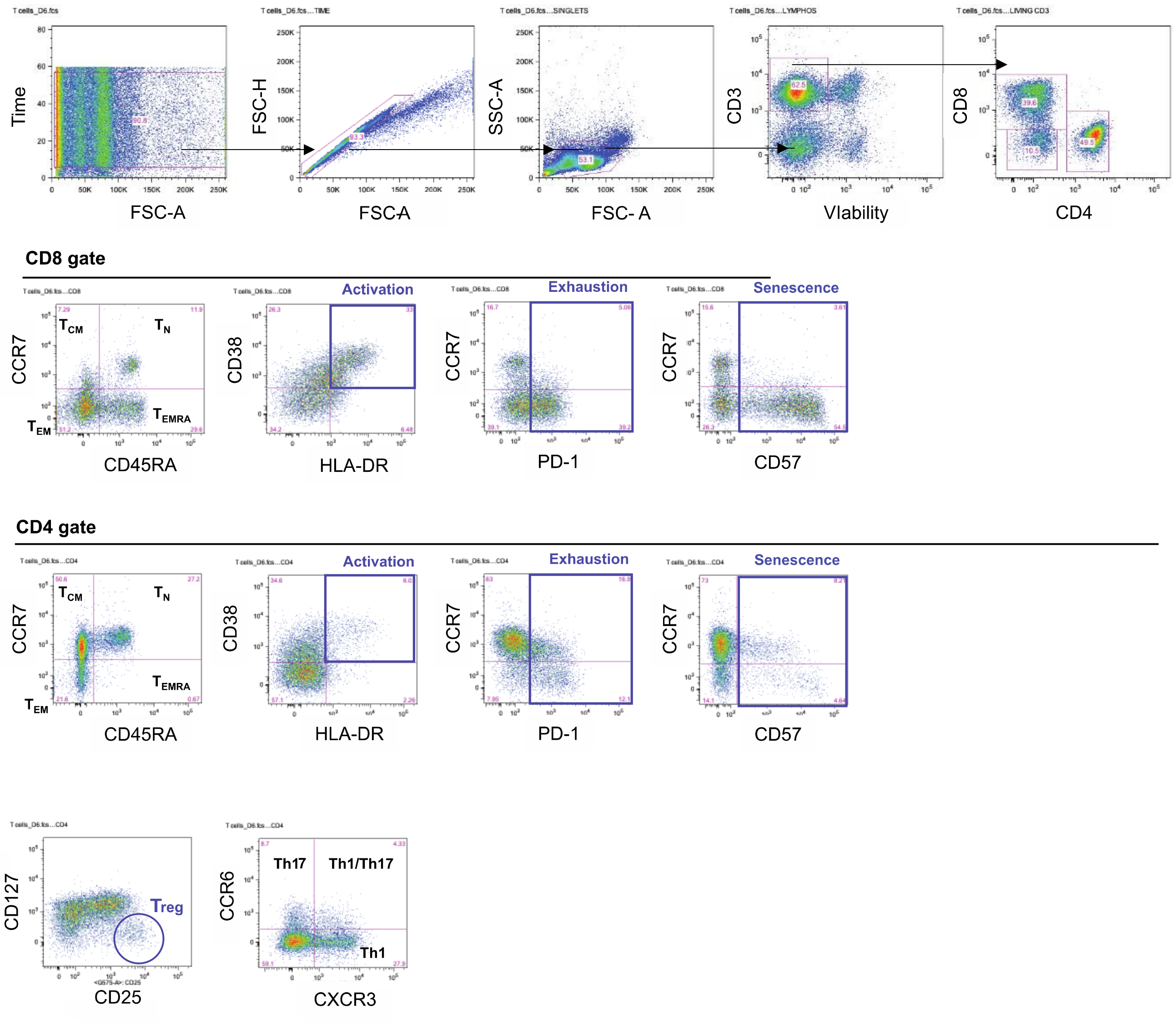

Supplement: Supplementary file 4 [file Image_2.PDF]
