# Supplementary material for: Dynamics of CD4 and CD8 T-Cell Subsets and Inflammatory Biomarkers during Early and Chronic HIV Infection in Mozambican Adults
Source: Front Immunol. 2018 Jan 5;8:1925. doi: 10.3389/fimmu.2017.01925 (PMC5760549; doi:10.3389/fimmu.2017.01925)

Dynamics of CD4 and CD8 T-cell subsets and inflammatory biomarkers during early and chronic HIV infection in Mozambican adults. **L Pastor *et al***

Supplementary Figure 3. CD4 T-cell activation along HIV infection.
Characterization of activated (A), exhausted (B), and senescent CD4 T-cells (C) across the different study groups and along time post-infection. M, months after infection. Box as IQR, middle line as median, whiskers as maximum and minimum and dots as individual observations.

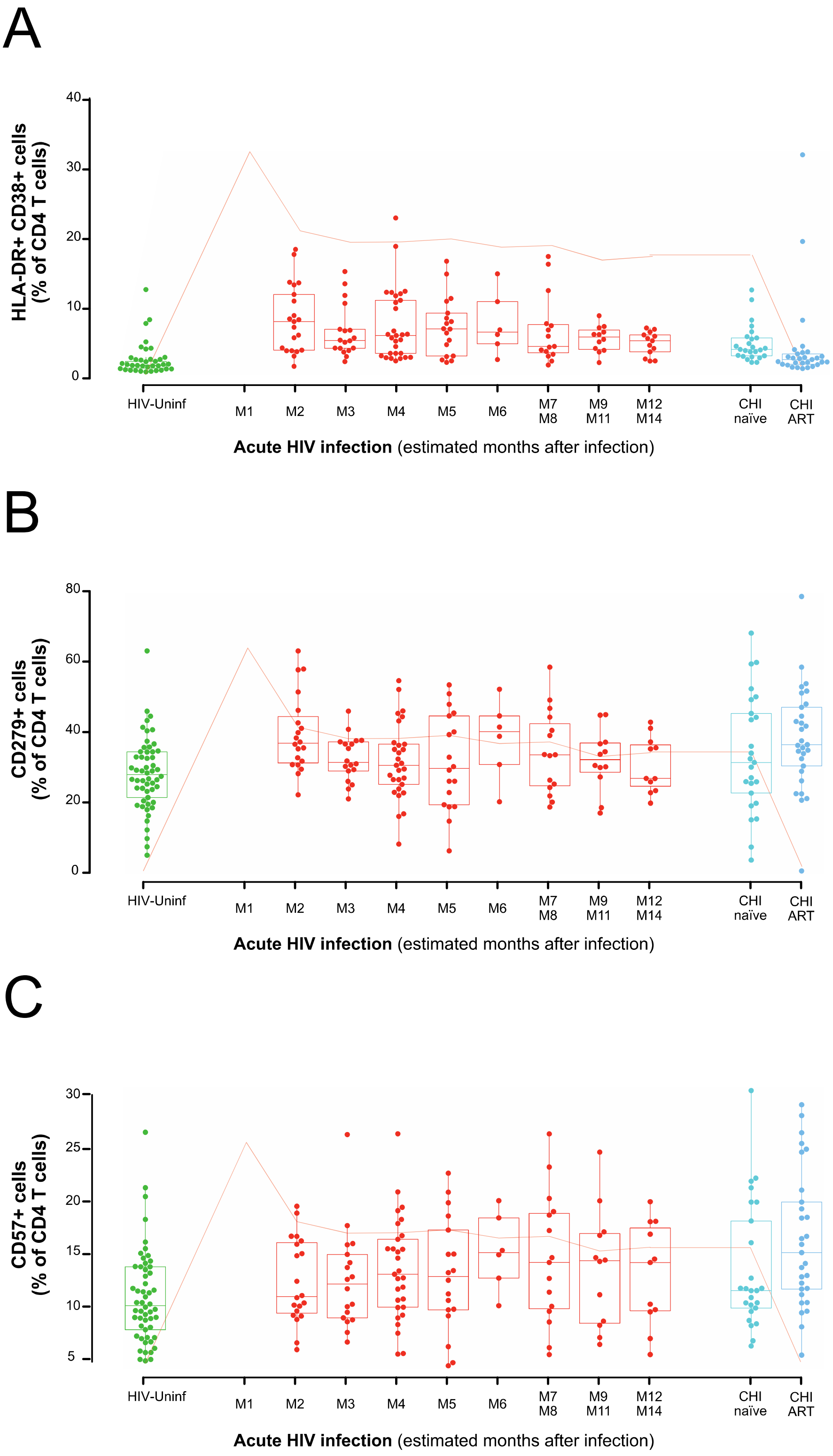

Supplement: Supplementary file 5 [file Image_3.PDF]
