# Supplementary material for: Dynamics of CD4 and CD8 T-Cell Subsets and Inflammatory Biomarkers during Early and Chronic HIV Infection in Mozambican Adults
Source: Front Immunol. 2018 Jan 5;8:1925. doi: 10.3389/fimmu.2017.01925 (PMC5760549; doi:10.3389/fimmu.2017.01925)

Supplementary Figure 4. Soluble biomarker dynamics along primary HIV infection. Individual profiles (grey lines) and fitted dynamics (red lines) are shown for the indicated soluble biomarkers.

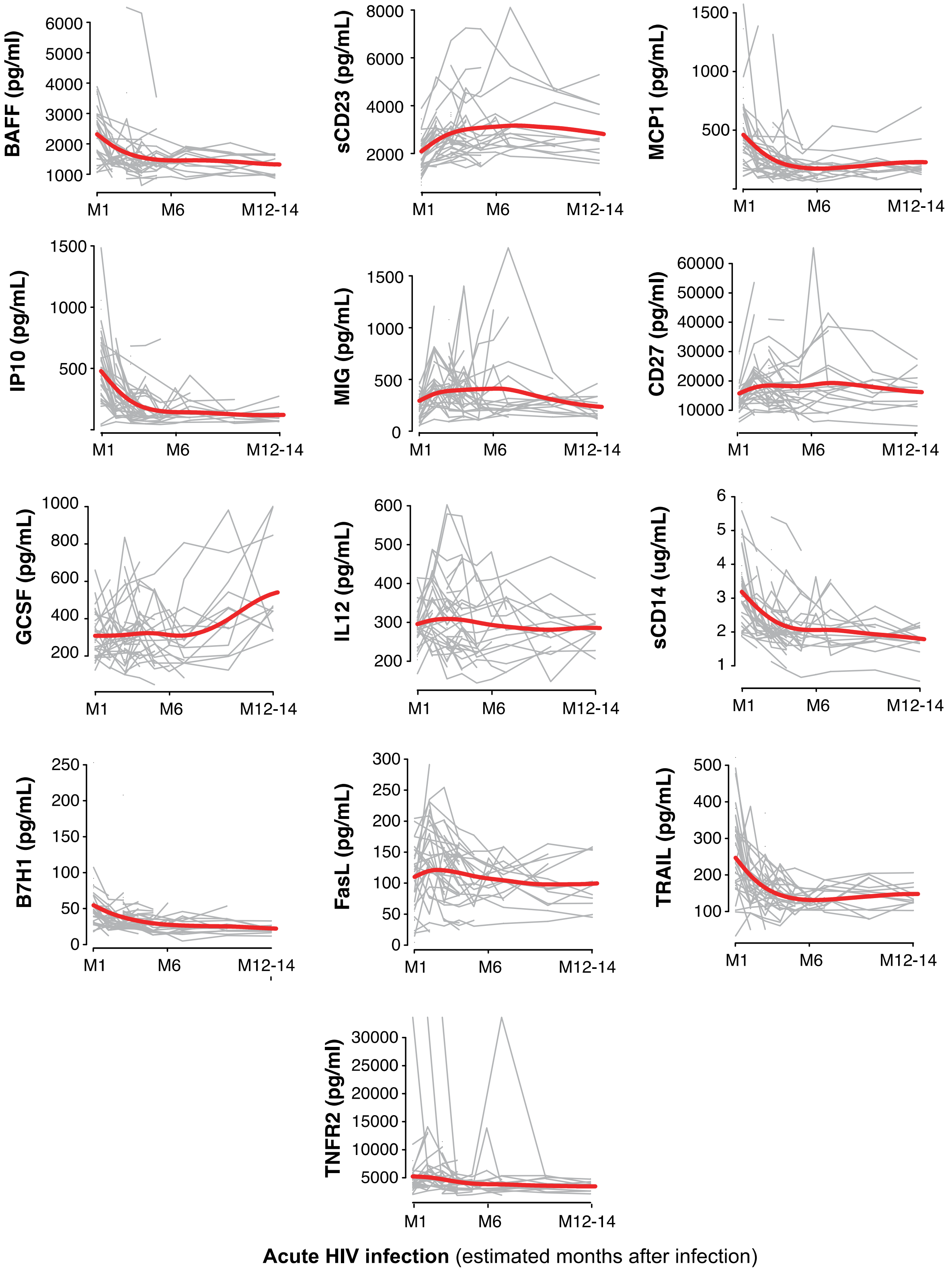

Supplement: Supplementary file 6 [file Image_4.PDF]
